# Biocompatibility, biodegradation, and antioxidative potential of alginate-dragon’s blood sap microbeads implanted subcutaneously in mice

**DOI:** 10.1590/acb414126

**Published:** 2026-07-24

**Authors:** Diego Armando Leite Carvalho, Gustavo Sousa Marques, Yanna Ribeiro Macêdo, Igor Iuco Castro-Silva

**Affiliations:** 1 Universidade Federal do Ceará – Postgraduate Program in Dentistry – Fortaleza (CE) – Brazil.; 2 Universidade Federal do Ceará – Dental School – Sobral (CE) – Brazil.

**Keywords:** Alginates, Biocompatible Materials, Oxidative Stress, Materials Testing

## Abstract

**Purpose:**

To evaluate alginate-dragon’s blood sap microbeads (DBSM) regarding biocompatibility, biodegradation, and antioxidative potential.

**Methods:**

Three DBSMs at concentrations of 1, 2, and 5% were developed by the drop spherification, and alginate-1% glycerin microbeads (GM) were used as control. Biocompatibility and biodegradation were evaluated by subcutaneous assay in 50 Swiss male mice, divided in the groups G1 (1% DBSM), G2 (2% DBSM) and G3 (5% DBSM), which were compared to C+ (1% GM) and C- (sham) at three and nine weeks. The histopathology of tissue irritation followed the ISO 10993-6 standard and the integrity of the biomaterials scored by quartiles. Metabolic analysis of intensity of catalase was carried out in the liver, kidneys, and tibias of the tested animals.

**Results:**

All microbeads were biocompatible, with less tissue irritation and greater partial resorption in G3 in subcutaneous tissue and preserved systemic antioxidant potential.

**Conclusion:**

The controlled release of dragon’s blood sap from *Croton lechleri* using alginate microbeads suggests a local anti-inflammatory action dependent on the concentration of the bioactive compound. Its role in repairing specialized connective tissues such as bone should be elucidated by future in-vivo orthotopic tests.

## Introduction

The sap of *Croton lechleri*, popularly known as dragon’s blood, is widely used in traditional Amazonian medicine, with applications mainly focused on anti-inflammatory, wound healing, antimicrobial and antioxidant action^1-3^. Its multi-action efficacy is justified by its diverse composition, with high concentrations of proanthocyanidins, terpenoids, phytosterols, saponins, polyphenols, flavonoids, other phenolic compounds, alkaloids, and protoalkaloids such as taspine^1,4^. Because its primary therapeutic use is topical, non-soluble dragon’s blood is poorly reported in the literature, with sap loading to calcium sulfate bone cement^5^, mesoporous silica nanoparticles^6^, chitosan or pullulan films^7^ or silk fibroin membrane^8^, or microencapsulated within whey protein concentrate and zein^9^.

Dental research on biomaterials for guided bone regeneration in Brazil over the last four decades has demonstrated advances in the use of polymers, aiming to generate biocompatible, bioresorbable products combined with bioactive compounds^10^. Economic factors also impact the development of the national biomaterials industry for bone regeneration, given the competitive prices of well-established foreign products and the strain on Brazilian consumer income^11^. The prospecting of biodiversity for developing *Croton lechleri* biomaterials strengthens technological sovereignty by providing high-performance alternatives^1,11^. This sustainable approach reduces dependence on imported inputs and promotes innovation in tissue engineering, aligning clinical demands with the preservation of regional flora^1,11^. Natural biopolymers associated with bioactives have attracted the production chain due to their sustainability, feasibility, and lower production costs^12-16^.

Alginate hydrogels are inexpensive, easy to handle, exhibit gelation in nanoporous networks with rapid diffusion of small molecules, contribute to the hydrophilicity of the extracellular matrix, and have good biocompatibility, making them attractive for applications in wound healing, drug delivery, and bone and vascular tissue engineering^13,17^. Non-enzymatic degradation of alginate occurs through dissolution with the release of divalent ions that crosslink the gel in the surrounding medium through exchange reactions with monovalent cations, such as sodium ions^13^. Microencapsulation with alginate beads can be applied to immobilize vegetable oils^12^, especially antioxidant phenolic compounds, as observed with rosemary^18^ and artichoke^19^. Thus, the local *in-vivo* administration of biologically active molecules would be slower and could have prolonged efficacy compared to direct injection without alginate encapsulation^20^.

All biomaterials need to undergo evaluation of their biological performance, which makes animal research extremely relevant and belonging to the base of the evidence pyramid^21^. Pre-clinical testing is necessary to validate biological safety, as well as their efficacy in generating some tissue stimulation, along with integrity compatible with the need and time for regeneration^22^.

This study evaluated alginate-dragon’s blood sap microbeads subcutaneously in mice regarding biocompatibility, biodegradation, and antioxidative potential.

## Methods

### Ethical and legal aspects

This study adopted the international principles of Replacement, Reduction and Refinement in Animal Research: Reporting of In Vivo Experiments or 3R-ARRIVE 2.0 guide^23^. The experimental protocol was approved by the Animal Use Ethics Committee of the Universidade Federal do Ceará, Sobral, CE, Brazil, under registration number 03/2023.

As the specie is not native to Brazil and was acquired commercially, there was no need for registration in the National System for the Management of Genetic Heritage and Associated Traditional Knowledge.

### Development of dragon’s blood sap microbeads

A 1.5% sodium alginate (Alquimia, Indaiatuba, SP, Brazil) diluted in distilled water (m/v) was used as the polymeric matrix, and 100 mL of solution was prepared in a beaker on a heated magnetic stirrer (OOH2O, Chicago, United States of America) for 40 minutes. After complete homogenization and waiting for another 40 minutes for oxygen elimination, it was added the 1% Tween 80 emulsion (Inlab, Sao Paulo, SP, Brazil) (v/v).

It was used as a bioactive dragon’s blood sap (Laszlo^TM^, Belo Horizonte, MG, Brazil), batch LZ2069, Brazilian National Health Surveillance Agency authorization no. 2.03740-2, process no. 25351.450246/2020-21, with an undetermined manufacturing date and expiration date in November 2026. From the pure form, it was possible to reach concentrations of 1, 2, or 5% (v/v) diluted in the heat-activated sodium alginate solution until acquiring a homogeneous and viscous hydrogel consistency, capable of flowing in a 1-mL syringe. As an inert control without bioactive, pure glycerin (Dinâmica, Indaiatuba, SP, Brazil) was used to produce the 1% concentration under the same protocol. The concentrations of 1, 2, and 5% were determined through preliminary pilot tests, as concentrations exceeding 5% compromised the solution’s viscosity for drip spherification, while concentrations below 1% were insufficient for analytical detection.

**Figure 1:**
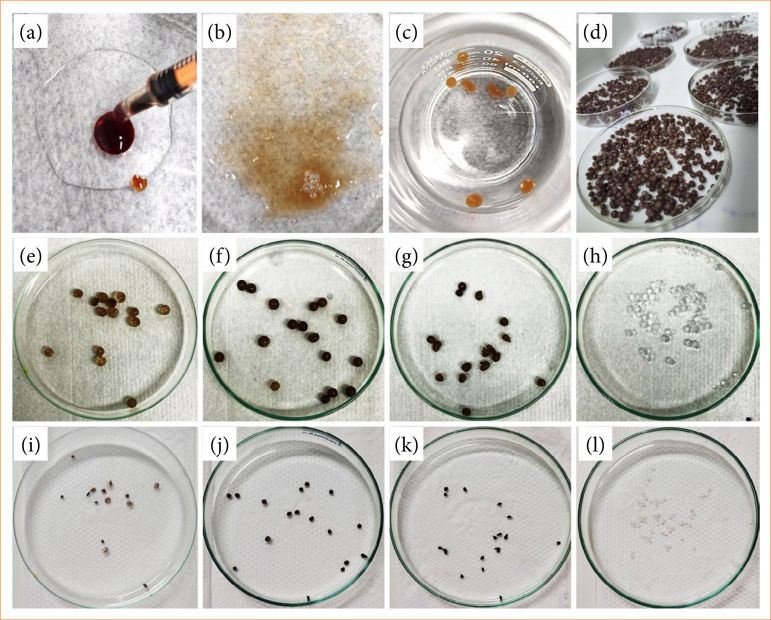
Development of dragon’s blood sap microbeads. (a) Mixture of *Croton lechleri* sap with sodium alginate, (b) homogenization, (c) spherification in calcium solution, (d) collection of microbeads, and beginning of the drying process for 1% dragon’s blood sap microbeads (DBSM), (f) 2% DBSM, (g) 5% DBSM, (h) 1% GM and (i–l) after 48 h.

Source: Elaborated by the authors.

The encapsulation of each mixture was performed by the direct method, *i.e.*, dripping into 100 mL of 1.5% calcium chloride solution (Alquimia, Indaiatuba, SP, Brazil) diluted in distilled water (m/v). The cross-reaction generated calcium alginate, a three-dimensional, insoluble, and stable gel network, causing immediate spherification of the initial mixture, and sodium chloride, a byproduct that remained dissolved. Continuous dripping produced multiple individual beads, dispersed in the solution. The drying phase at room temperature for 48 hours showed optimal conditions in the spheroidal and uniform pattern for all proposed concentrations for dragon’s blood sap microbeads (DBSM) and glycerin microbeads (GM). The spheres underwent advanced syneresis, evidenced by a reduction in volume to one-fifth for 1% DBSM and 1% GM and up to one-third of the original for 2 and 5% DBSM, stabilizing their micrometric shape below 1 mm in diameter after this period. There was a progressive dark brown coloration, increasing with the concentration of the bioactive, unlike the GM, which maintained their characteristic translucency (Fig. 1).

All dried plastic forms were considered stable, allowing good handling and ultraviolet (UV) radiation sterilization in a UV Box Pro chamber (ELG, Joinville, SC, Brazil) for the *in-vivo* study.

### Subcutaneous implantation

**Figure 2:**
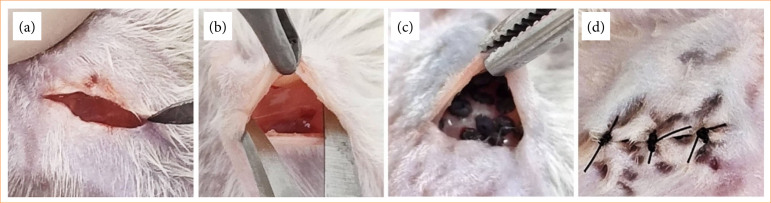
Surgery for placing dragon’s blood sap microspheres into the subcutaneous tissue of mice. (a) Incision, (b) divulsion, (c) implantation, and (d) suture.

Source: Elaborated by the authors.

The subcutaneous bed was chosen to evaluate the preliminary response in the host connective tissue in a controlled manner to the developed biomaterials. The experimental model used was the heterogenic albino Swiss mouse (*Mus musculus*), male, young adult, with an average weight of 30 g. The animals remained in collective cages according to the experimental group, in a climate-controlled vivarium, with a 12-hour light/dark cycle, food and water *ad libitum*. Fifty animals were distributed according to different experimental conditions, considering five groups, two times and five specimens each. Intraperitoneal anesthesia was administered with 10% ketamine solution (Dopalen, Sespo Indústria e Comércio LTDA, Paulínia, SP, Brazil), at a dose of 80 mg/kg, and 2% xylazine (Anasedan, Sespo Indústria e Comércio LTDA, Paulínia, SP, Brazil), at a dose of 10 mg/kg. Trichotomy of the trunk-dorsal region was performed, and antisepsis was performed with 0.5% aqueous chlorhexidine. A 1-cm linear incision was made, followed by tissue divulsion to form a subdermal pocket. Each animal received a subcutaneous implant corresponding to groups G1 (1% DBSM), G2 (2% DBSM), G3 (5% DBSM) or C+ (1% GM) or remained without implant (C-), only with the surgical bed filled with blood clot or sham group. Then, the operated regions received simple sutures with mononylon 4.0 thread (Procare, Medico Industries & Trade Co-Shijiazhuang, China). In all groups and experimental times, the presence of postoperative infection was not observed (Fig. 2).

### Data collection and analysis

After three and nine weeks, the animals were euthanized by an overdose of the anesthetic solution (three times the usual dose), and an immediate excisional necropsy of the skin area deepened at the level of the subcutaneous procedure was performed. In addition, the kidneys and liver were also removed for study, as they are organs linked to metabolic biotransformation, and the tibias, as the largest bones in mice and suitable for investigating bone behavior.

All samples from the subcutaneous bed were fixed in 10% (v/v) buffered formalin solution, pH 7.0, for 48 h. After fixation, the necropsies were washed in running water for 1 h, cleaved longitudinally, dehydrated in increasing baths of 70 to 100% ethanol, bathed in xylene, impregnated and embedded in paraffin. The paraffin blocks were microtomized in 4-µm sections and stained with hematoxylin-eosin (HE). All histological slides were examined under the supervision of an experienced pathologist.

To characterize biocompatibility and biodegradation, a qualitative and quantitative analysis was conducted. Five images of each sample were captured in adjacent, non-overlapping fields in the center of the implant or surgically manipulated area along its entire length, using a biological microscope DI-260 (Digilab, Piracicaba, SP, Brazil) attached to a digital camera model NewValue 48 Megapixel (Panasonic, São Paulo, SP, Brazil). For qualitative analysis, slides from each experimental group were selected and morphologically described to represent the observed events.

Quantitative analysis of biocompatibility or irritation pattern adopted the guidelines of ISO 10993-6^24^, considering the presence of neutrophils, lymphocytes, macrophages, and foreign body giant cells as inflammatory criteria, while the presence of neovascularization and connective tissue as reparative criteria with a total magnification of 400x. The standard assesses the presence of such criteria by scores defined as 0 (absent), 1 (rare), 2 (moderate), 3 (intense), or 4 (overcrowding), generating a numerical system capable of determining the irritation pattern. To determine the irritation pattern of each condition (25 results, considering quintuplicates of animals and images of each test and control group), three equations were used: Eq. 1, for the inflammation pattern (*I*_*x*_); Eq. 2, for repair pattern (*R*_*x*_); and Eq. 3, for irritation pattern:


(1) $$I_{x}=2\sum (Nt+L+M+FBGC)$$

where: *I*_*x*_: inflammation pattern by test or control group; *Nt*: mean neutrophil score; *L*: mean lymphocyte score; *M*: mean macrophage score; *FBGC*: mean foreign body giant cell score.


(2) $$R_{x}=\sum (Nv+CT)$$

where: *R*_*x*_: repair pattern by test or control group; *Nv*: mean neovascularization score; *CT*: mean connective tissue score.


(3) $$IP_{x}=(I_{x}+R_{x})-(I_{C-}+R_{C-})$$

where: *IP*_*x*_: total irritation pattern by test group or positive control; *I*_*x*_: mean inflammation pattern score by group; *R*_*x*_: average repair pattern score by group; *I*_*C-*_: mean score of inflammation pattern in negative control; *R*_*C–*_: average score of the repair pattern in the negative control.

After the general calculations, the ISO 10993-6 standard adopts negative control as the standard for the other groups, by subtracting it from the others, to reduce interpretation bias. Finally, the actual irritation pattern of the experimental groups corresponds to one of the following ranges: non-irritating (0.0–2.9), mildly irritating (3.0–8.9), moderately irritating (9.0–15.0), or severely irritating (> 15). A negative result is considered standard 0.

To histologically analyze biodegradation in the photomicrographs obtained with a total magnification of 40x in each test and control group, scores from 0 to 4 was proposed in to grade the integrity or presence of the biomaterial by quartiles, defined as 0 (absent), 1 (minimal: up to 25%), 2 (mild: up to 50%), 3 (moderate: up to 75%) or 4 (predominant: above 75%). The raw data were tabulated in Excel (Microsoft Office, United States of America), expressed graphically as mean ± standard deviation, and statistically analyzed using Jamovi software version 2.3.28 (Jamovi Project, Australia) for comparisons between groups according to the criteria and experimental times.

Functional study was then conducted with the left kidney, tibia, and smaller lobe of the liver, intended for biochemical analyses. Fresh samples were kept frozen at -20°C until the time of the experiments, when they were used at room temperature. A quantitative analytical technique was performed using catalase test to identify the preservation of the antioxidant reaction of native tissue to hydrogen peroxide. Each fragment of 4 mm in diameter and 1 mm in thickness obtained by punch and scalpel was placed in individual 1-mL wells, dripped with 200 µL of 3% hydrogen peroxide solution (Millipore Sigma, United States of America), and the effect of active endogenous catalase, dissociating hydrogen peroxide into oxygen gas and water, was observed at room temperature for 15 seconds for liver and kidney and 30 seconds for bone. The results were categorized according to the intensity of bubbling in the samples studied as 0 (absent), 1 (weak), 2 (moderate), and 3 (strong). All tests were interpreted by two examiners in agreement and supervised by an experienced pathologist.

The data distribution was subjected to the Shapiro-Wilk’s test and once its non-normality was confirmed, the non-parametric Kruskal-Wallis’ test and the Dwass-Steel-Critchlow-Fligner multiple comparisons *post hoc* test were applied, considering a 95% confidence interval and significant differences if *p* < 0.05.

## Results

### Major morphological aspects

**Figure 3:**
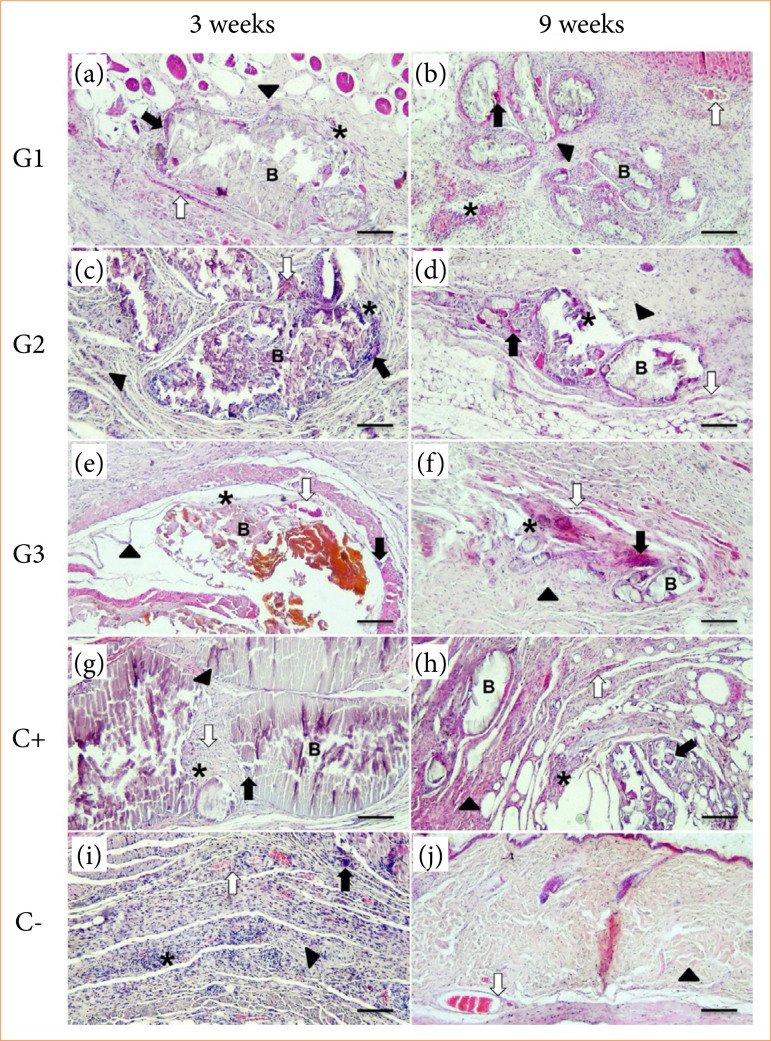
Histological analysis of biocompatibility and biodegradation of alginate-dragon’s blood microbeads of (a and b) G1, (c and d) G2 and (e and f) G3 or (g and h) glycerin microbeads of C+ implanted subcutaneously in mice and sham group or (i and j) C- at three and nine weeks. Staining: hematoxylin-eosin. Scale bar: 100 μm.

All microbeads were spheroids, with diameters between 500 and 600 µm, and translucent like alginate, with increasing hue according to the higher concentration of dragon’s blood, being chromophobic in G1, pink in G2, and partially reddish orange in G3. There was a surrounding and variable presence of inflammatory cells, connective tissue, and blood vessels when compared to C+ and C- at three weeks. At nine weeks, a homogeneous staining pattern like C+ was observed, suggesting complete release of the bioactive compound in G2 and G3. The microbeads underwent disintegration due to the permeation of inflammatory cells and connective tissue within their internal structure, generating multiple and reduced fragments at the end of the experiment. For DBSM, the diameters varied considerably between 50 and 200 µm, with G3 showing the fewest fragments, while C+ exhibited implant remnants up to 300 µm. Furthermore, G3 was closer to G- in reducing inflammation and advancing repair compared to G2, while G1 and C+ showed more delayed results associated with the inflammatory phase. Neither group exhibited a dense fibrous capsule, characteristic of a response to a foreign body (Fig. 3).

B: biomaterial; *inflammatory infiltrate; black arrow: giant cell; white arrow: blood vessel; arrowhead: fibrosis. Source: Elaborated by the authors.

### Biocompatibility

**Figure 4:**
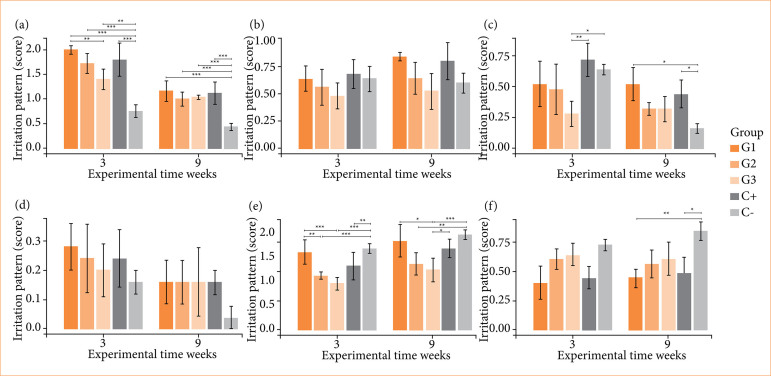
Intensity of biocompatibility criteria for microbeads and controls implanted subcutaneously in mice after three and nine weeks: (a) neutrophils, (b) lymphocytes, (c) macrophages, (d) giant cells, (e) fibrosis, and (f) neovascularization.

In the quantitative histological analysis, the groups exhibited heterogeneous responses. Neutrophil counts for all biomaterials were higher than C- at three and nine weeks, and lower in G3 compared to G1 at three weeks. Lymphocyte counts did not differ between groups at three and nine weeks. Macrophage counts were lower in G3 compared to C+ and C- at three weeks, while at nine weeks G1 and C+ were higher than C-. Multinucleated giant cell counts did not differ between groups at three and nine weeks, but this criterion showed the greatest intragroup variance. Fibrosis count was lower at three weeks for G2 and G3 compared to G1 and C-, and for C+ compared to C-, while at nine weeks it was lower for G2 compared to C- and for G3 compared to G1, C+, and C-. Neovascularization counts did not differ between groups at three weeks, but G1 and C+ were lower than C- at nine weeks. There was a general trend of decreasing inflammatory phenomena and increasing reparative phenomena between three and nine weeks, while there was a trend among treatments of decreasing intensity of the criteria according to the higher concentration of the bioactive ingredient dragon’s blood. G3 performed better, with a lower frequency of neutrophils and macrophages at three weeks and fibrosis up to nine weeks, more like C- in the reaction of lymphocytes, giant cells, and neovascularization throughout the experimental period (Fig. 4).

*p = 0.01–0.04; **p = 0.001–0.009; ***p < 0.001. Source: Elaborated by the authors.

The overall irritation pattern showed greater reactivity in the microbeads of groups G1 and C+ compared to G2 and G3, with significantly lower scores at three and nine weeks. Over time, there was a trend towards a slight increase in irritation in G1, G3, and C+, but the low scores allowed all groups to be classified as non-irritating. G3 achieved the lowest irritation scores, especially due to presenting inflammatory criteria with the lowest scores among the implanted microbeads (Table 1).

**Table 1:** Irritation pattern of alginate-dragon’s blood sap or glycerin microbeads in subcutaneous tissue in mice at three and nine weeks.

Experimental times (weeks)	Experimental groups			
	G1	G2	G3	C+
Three	2.08 (NI)	0.92 (NI)	0.00 (NI)	1.84 (NI)
Nine	2.36 (NI)	0.88 (NI)	0.64 (NI)	1.92 (NI)

G1: alginate-1% dragon’s blood sap microbeads (DBSM); G2: 2% DBSM; G3: 5% DBSM; C+: alginate-1% glycerin microbeads; NI: non-irritating. Source: Elaborated by the authors.

### Biodegradation

**Figure 5:**
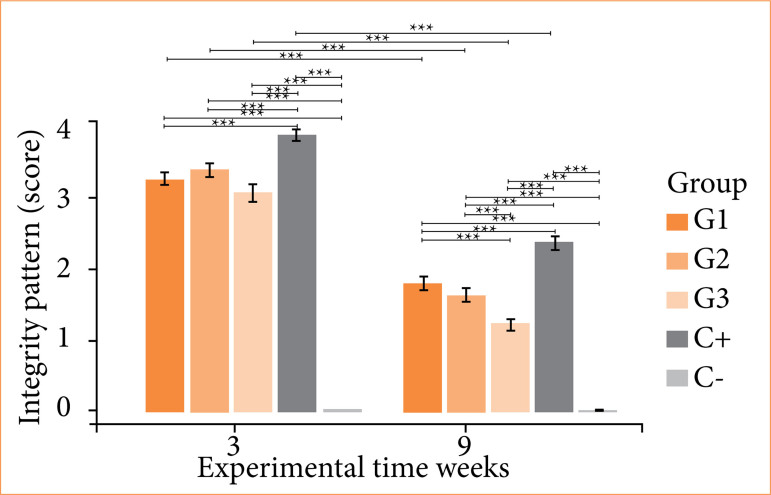
Intensity of biodegradation for microbeads and controls implanted subcutaneously in mice after three and nine weeks.

The integrity of the biomaterials did not vary between groups G1, G2, and G3 at three weeks, but these groups had reduced scores compared to C+. At nine weeks, differences were observed between all groups, with greater degradation in G3, followed by G2, G1, and C+. Comparing the experimental times of three and nine weeks, it was possible to verify that all biomaterials were partially degraded. Thus, the greatest degradation was related to the higher concentration of dragon’s blood, which proved to be more sensitive for nine weeks (Fig. 5).

***p < 0.001. Source: Elaborated by the authors.

### Antioxidative potential

**Figure 6:**
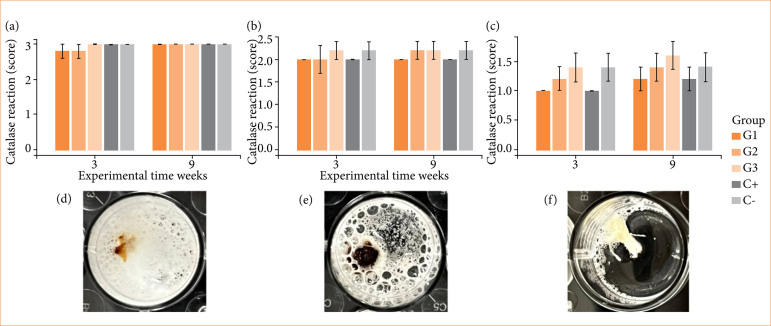
Analysis of the antioxidant potential using catalase reaction for (a) liver, (b) kidney and (c) tibia. Strong intensity for (d) liver, (e) moderate intensity for kidney and (f) weak intensity for tibia.

Source: Elaborated by the authors.

Despite a suggestive mild and progressive improvement from three to nine weeks in the groups treated with DBSM, especially for G2 and G3 compared to G1 and C+, and in the tibia samples, there were no significant differences between the groups compared to C-. There was greater catalase activity in the liver with a median score of 3, compared to the kidneys with a median score of 2, and the tibia, with a median score of 1 for all experimental conditions. Thus, all implanted microspheres preserved the same systemic antioxidant potential as the sham condition (Fig. 6).

## Discussion

The development of this controlled-release formulation is justified by the chemical instability of *Croton lechleri* sap in its pure form. Although readily accessible, the raw sap is highly susceptible to rapid oxidation and polymerization upon exposure to environmental factors and biological fluids, which significantly narrows its therapeutic window^25^. Consistent with the parameters discussed for microbead formation, encapsulating the sap within an alginate matrix serves as a strategic protective barrier, shielding sensitive bioactives—such as taspine—from premature degradation. Furthermore, the transition from a liquid resin to the solid microsphere system described in our protocol optimizes clinical handling and ensures precise localization at the surgical site. This biotechnological approach contributes to the stability of the natural bioactive compounds against adverse conditions^9,26^.

Sodium alginate appears to be the most critical factor for the quality of microbeads formation. Ideal conditions for encapsulating liquids include 2% alginate with a hardening time of 10 min^18^, 2.25% alginate and 2 min gelation time^19^, or a range varying from 1.5 up to 2.5% alginate, which produced spheres up to 1 mm in diameter^16^. Our protocol was similar to Paula’s et al.^16^, including a calcium chloride solution, Tween 80, without prior purification and dried for 48 hours, which doped *Croton zehntneri* essential oil from 1.2 to 10.7, with 57% release of the bioactive compound after 4 hours and a 35% mass loss of alginate spheres after 70 days *in vitro*. The lower concentration of 1.5% sodium alginate may be justified by the higher density of the dragon’s blood sap of *Croton lechleri*, a parameter similar to that used by Santos et al.^27^, who encapsulated hydroxyapatite and obtained microspheres in the granulometric ranges of 250–425 and 425–600 μm, with the smaller ones being more favorable for the regeneration of bone defects in the calvaria of Wistar rats due to the better framework for filling. Therapeutic approaches using *Croton lechleri* have ranged from unidentified concentrations in carious lesions^28^ to specific variations, such as 2.5% for rabbit dental pulp^29^, and 10% for human periodontal ligament preservation^30^. In this context, our formulations between 1% and 5% sit within an effective therapeutic range while providing the added advantage of a controlled-release matrix.

*In-vivo* response to implants demonstrates biological variations, with a prevalence for acute inflammation of neutrophils between three and 10 days, for chronic inflammation of lymphocytes and macrophages between 10 and 60 days, and giant cells between 15 and 60 days, while repair involves fibroplasia between 10 and 15 days, neovascularization between 10 and 60 days, and fibrogenesis up to 60 days^31^. The composition or processing of biomaterials elicits different reactions in the subcutaneous tissue of mice. Alginate-capsul polymers show a discreet presence of multinucleated giant cells and the beginning of neovascularization after three weeks, and after nine weeks, an abundant presence of loose connective tissue and persistence of a moderate mononuclear inflammatory reaction, which differs from the pattern of collagenous biomaterials^32^. The controlled release of dragon’s blood sap using alginate as a carrier suggests local anti-inflammatory action, and its antifibrotic and pro-angiogenic stimuli may be promising strategies in the repair of specialized connective tissues.

Considering the irritation pattern, the collagen-apatite-nanokeratin composite between three and nine weeks was mildly irritating (3.72 and 3.00, respectively) compared to pure collagen, which was non-irritating (0.00) in both periods^33^. Composites with a higher collagen gelatin content (80%) ranged from mildly irritating (3.76) at three weeks to non-irritating (2.60) at nine weeks, while those with a higher apatite content (40%) remained mildly irritating at three (6.64) and nine weeks (4.08)^34^. As an alternative to collagen matrices, cellulose-apatite composites with the bioactive strontium generated moderate irritation between three and nine weeks, with a low inflammatory response and an increase in connective tissue repair, showing temporal differences between non-oxidized (3.40 and 3.72) and oxidized (7.68 and 8.48) formulations^14,22^. Based on these data, the performance obtained with DBSM was excellent, due to the lack of irritation observed in all groups and, especially, in higher concentrations of *Croton lechleri*, thus confirming its bioactivity.

Oral administration of lyophilized latex of *Croton lechleri* Muell. Arg. did not generate toxicity in the hematological and biochemical parameters studied in *Rattus norvegicus* var albinus^35^. In Wistar rats treated with topical application of *Croton lechleri* on skin wounds, re-epithelialization and granulation tissue rich in fibroblasts and blood vessels were observed after seven days, while the control group exhibited a fibrinoleukocytic crust and intense inflammation in the connective tissue, with areas of abscesses, granulomas, and necrosis^36^. The assistance in wound repair with epithelial contraction and new collagen formation could be related to 3’,4-O-dimethylcedrusin and taspine present in pure Croton dragon’s blood, as observed microscopically over 10 to 30 days^37^. Campos^38^ implanted polyethylene tubes containing *Croton lechleri* sap into the subcutaneous tissue of rats and observed after seven days an inflammatory response of macrophages and lymphocytes similar to the control, after 15 days a differentiated connective tissue, well vascularized and with fewer inflammatory cells than the control, and after 30 days, well-developed connective tissue rich in collagen fibers, a small number of blood vessels and some lymphocytes, being considered biocompatible. It has been found that taspine increases fibroblast migration, and this could explain the accelerated fibrogenesis^4^.

On the other hand, dragon’s blood from *Croton lechleri* showed immunomodulatory activity, inhibiting the proliferation of activated T-cells^39^. Furthermore, *Croton lechleri* exhibited anti-collagenase activity with a greater inhibitory potential than the positive control epigallocatechin gallate, explained by the presence of catechins, a type of flavonoid/polyphenol typical of its composition^40^. This modulating behavior in connective tissue is particularly interesting and has repercussions in clinical applications, as observed by Martins et al.^30^, who used dragon’s blood sap from *Croton lechleri* to preserve the viability of periodontal ligament cells. According to our results, low doses of dragon’s blood appear to contribute more to promoting the fibrogenic pathway.

Glycerol is crucial for water retention and for improving skin elasticity in aquaporin-3-deficient mice^41^. Paca peritoneal membrane preserved in 98% glycerin demonstrated lower malleability and greater rigidity and when implanted in the abdominal wall of Wistar rats histologically, it showed large inflammatory infiltrate in seven and 15 days, with frequent adverse effects such as seroma and fistula, and a large presence of connective tissue in 30 and 60 days, ensuring good healing^42^. Paula et al.^16^ conjugated glycerin in alginate formulations and higher concentrations of the encapsulating solution generated microspheres with larger diameter and weight, which can be justified by its plasticizing effect. These findings suggest that glycerol may have a dual effect on connective tissue, acting primarily as a moisturizing agent, but causing an increase in passive stiffness, or fibrosis, in some applications, with the effect depending on the concentration and method of application.

Resorbable biomaterials generally undergo intense disintegration between 30 and 60 days, although different chemical processes can extend this period to extend the functional stability of the scaffold^31^. Although desirable, the degradation of polymers with a lower resorption rate can increase the irritation pattern, especially by stimulating giant cells and mononuclear infiltrate^14,22,32^. Despite a slight tendency toward an increase in the overall score, the analyzed groups maintained the pattern of non-irritating biomaterials as they underwent partial resorption. Processes involving oxidation can make cellulosic biomaterials more susceptible to significant degradation starting at three weeks in the subcutaneous tissue of mice, with half the original volume reduced at nine weeks^22^. On the other hand, crosslinking collagen-based hydrogels or adding apatites would help prolong their integrity, as they undergo more intense degradation after nine weeks in the subcutaneous tissue of mice^33,34^.

The latex from *Croton lechleri* can stimulate phagocytosis via a direct concentration-dependent pathway, with lower concentrations stimulating greater phagocytic capacity in human monocytes than in human neutrophils, while even at higher latex concentrations, the stimulation of phagocytosis in murine macrophages-monocytes fell short of that in other cell lines^39^. This suggests that the release of dragon’s blood could accelerate the degradation of the microbeads since biomaterials containing alginate undergo only partial and slow resorption^32^. Our results corroborate this evidence, with partial degradation of the microbeads becoming more evident with higher concentrations of dragon’s blood, although conversely, a decrease in the density of phagocytic cells was observed. The period between three and nine weeks appears to have been more critical for digestive events mediated by macrophages and giant cells^31^, to explain the earlier biodegradation induced by the bioactive compared to the control. The use of degradable hydrogels featuring norbornene-modified alginate crosslinked with two different thiol-coupled peptides susceptible to cleavage by matrix metalloproteinases (MMPs) could favor, after eight weeks in subcutaneous implants in C57/Bl6 mice, a more significant rate of cellular infiltration and areas with fibers reaching twice that of the untreated control for enzymatic action^17^. However, it is important to note that even if the gel dissolves, the average molecular weights of many commercially available alginates are higher than the renal clearance threshold, and they are unlikely to be completely removed from the body^13^.

Studies claim that hydrophilic polymers injected subcutaneously into mice, such as polyethylene glycol, follow a distant distribution predominantly in the kidneys, but also in the heart, lungs, and liver^43^. It is reported that, depending on the concentration of *Croton lechleri*, there may be antioxidant or prooxidant activity by neutrophils, monocytes, and lymphocytes in response to H_2_O_2_^39^. *Croton lechleri* sap (1 and 10 μg/mL) was shown to significantly decrease H_2_O_2_-induced reactive oxygen species production in human umbilical vein endothelial cells compared to baseline^44^. Antioxidant activity of dragon’s blood of *Croton lechleri* was tested using hydroperoxide-initiated chemiluminescence in rat liver homogenates, and the latex showed an increase in light emission, suggesting the presence of prooxidant compounds at higher concentrations compared to the control^45^. Even with the expected biotransformation, no change in reactivity was observed in the kidneys, liver, or tibia, unlike Costa et al.^22^, who observed a progressive increase in renal catalase from three to nine weeks and exhibited a higher mean bone catalase reactivity until nine weeks for the bioactive strontium conjugated to apatite and cellulose. Our study focused on evaluating catalase, found within cellular organelles such as peroxisomes and mitochondria, for a systemic overview of its antioxidant potential, but more specific enzymatic or non-enzymatic studies could contribute to understanding tissue-dependent protection against free radicals^46^.

Despite the Amazon’s abundance of natural resources that are sources of bioactive compounds, it has a low level of technological readiness (TRL), which makes the Brazilian market less competitive than the United States of America’s market^47,48^. Our study sought to mitigate this scenario and fell within the prototype and initial animal testing phase, consistent with a TRL 4, prior to the validation and production phases. Regarding industrial scalability, although the manual drip-spherification method was effective for this initial characterization, it presents limitations for large-scale production. A viable technological adaptation for industrial purposes would be the Coaxial Air-Jet System. In this process, the bioactive flows through a central nozzle while a compressed air stream in the outer nozzle facilitates droplet formation, providing precise control over bead size and high throughput^47^.

The impact of this study lies in filling the gap in biological research on dragon’s blood and its methods of administration via implant for tissue regeneration^1,2^. Furthermore, it contributes to cost-effective proposals applied to real-world demands, aiming to foster the development of Brazilian biotechnology in a context of still pronounced inequalities and chronic underfunding^49^. Tests on critical defects and their correlation with other carriers are also encouraged to unveil their potential in bone metabolism^15^.

## Conclusion

Alginate microbeads with dragon’s blood sap from *Croton lechleri* were biocompatible and partially bioresorbable in subcutaneous tissue and preserved systemic antioxidant potential in the liver, kidney, and tibia of mice. The controlled release using alginate microbeads suggests a local anti-inflammatory action dependent on the concentration of the bioactive compound. Ensuring commercial viability requires further studies on the long-term stability and shelf-life of the dried microbeads. Its role in repairing specialized connective tissues such as bone should be elucidated by future in-vivo orthotopic tests.

## Data Availability

All datasets were generated or analyzed in the present study.
